# Supplementary material for: Evolutionary dynamics of cancer in response to targeted combination therapy
Source: eLife. 2013 Jun 25;2:e00747. doi: 10.7554/eLife.00747 (PMC3691570; doi:10.7554/eLife.00747)
Supplement: Supplementary file 1. — DOI: http://dx.doi.org/10.7554/eLife.00747.012 [file elife00747s002.pdf]

Evolutionary dynamics of cancer in response to targeted combination therapy
Supplementary File 1: Mathematical Proofs

Bozic et al.

1 Notation

1.1 Resistance profiles

Consider a cancer therapy consisting of D targeted drugs, indexed $i = 1, \ldots, D$. The resistance properties of each tumor cell can be summarized by its *resistance profile*, a binary string of length D, with 1's indicating which drugs the cell is resistant to. For example, the resistance profile 010 indicates that a cell is resistant to the second of three drugs, but sensitive to the first and third. The tumor is initiated by cells of type $0 \ldots 0$—sensitive to all drugs. Other profiles arise through resistance mutations. We let n_i denote the number of point mutations that would confer resistance to drug i. We also allow for the possibility that one point mutation may confer resistance to multiple drugs. The number of point mutations that would confer resistance to drugs $i_1, \ldots, i_m$ (but not to the other $D - m$ drugs), with $1 \leq i_1 < \ldots < i_m \leq D$, is denoted $n_{i_1 \ldots i_m}$. We disregard the possibility of losing drug resistance through mutation. Also, while our notation reflects an assumption that drug resistance arises via single point mutations, our results can readily be applied to situations in which multiple mutations are required for resistance to a single drug.

1.2 Branching process model

As described in the main text, we model the evolution of resistance as a stochastic branching process, in which each resistance profile is identified as a distinct type. Prior to treatment, all cell types divide at rate b and die at rate d. Thus the tumor expands at rate $r = b - d$ prior to treatment. Treatment is initiated when the tumor has reached a detection size of M cells. During treatment, we suppose that cell types sensitive to at least

one drug (i.e. those with resistance profiles other than $1\dots1$) divide and die at rates b' and d', respectively, with $r' = b' - d' < 0$. Fully resistant cells (those with profile $1\dots1$) continue to divide and die at rates b and d, respectively. (More generally, one could suppose that each resistance profile has distinct birth and death rates during treatment, but we do not consider such generality here.)

With each reproduction, one of the daughter cells acquires each potential point mutation with probability equal to the point mutation rate u. (Our model assumes that only one of the daughter cells can acquire mutations, which amounts to rescaling the mutation rate by a factor of two.) Thus, for each combination of drugs $i_1, \dots, i_m$, the probability that one of the $n_{i_1 \dots i_d}$ resistance mutations occurs in a daughter cell is $(1-u)^{n_{i_1 \dots i_m}} \approx 1 - n_{i_1 \dots i_m} u$. (This approximation assumes that $u n_{i_1 \dots i_m} \ll 1$—that is, resistance mutations are rare—so that the possibility of multiple resistance mutations in a single reproduction event can be disregarded.)

1.3 Paths to full resistance

We are most interested in the emergence of simultaneous resistance to all D drugs, leading to treatment failure. Such multi-drug resistance may occur via multiple paths, where a *path* is a sequence of resistance mutations leading from $0\dots0$ (no resistance) to $1\dots1$ (resistance to all drugs). An example for three drugs is the path $000 \to 010 \to 111$ (resistance to drug 2 first, then 1 and 3 simultaneously). Each path involves some number m of mutations, $1 \le m \le D$. Along a path there are $m+1$ resistance profiles, indexed $j = 0, \dots, m$. The initial resistance profile $0\dots0$ is indexed $j = 0$, while the final, $1\dots1$, is indexed $j = m$. The number of potential point mutations that would lead from profile $j-1$ to profile j, along a particular path, is denoted ν_j. The number of potential point mutations that would lead from profile $j-1$ to a profile not on the path in question is denoted η_j. In short, ν_j and η_j are the numbers of "on-path" and "off-path" mutations, respectively, from profile $j-1$. So, for example, for the path $000 \to 010 \to 111$ we have

$$\begin{aligned}
\nu_1 &= n_2 \\
\eta_1 &= n_1 + n_3 + n_{12} + n_{13} + n_{23} + n_{123} \\
\nu_2 &= n_{13} + n_{123} \\
\eta_2 &= n_1 + n_3 + n_{12} + n_{23}.
\end{aligned}$$

Thus, with each division of a cell of profile $j-1$, we have the following probabilities of events (assuming $(\nu_j + \eta_j)u \ll 1$, i.e. resistance mutations

are rare):

$$\begin{cases} 1-(\nu_j+\eta_j)u & \text{two daughters of profile } j-1 \\ \nu_j u & \text{one daughter of profile } j-1 \text{ and one of profile } j \\ \eta_j u & \text{one daughter of profile } j-1 \text{ and one off-path daughter.} \end{cases}$$

1.4 The rare-mutation, large-tumor-size limit

In human cancers, the point mutation rate is very small ($u \sim 10^{-9}$) and the number of cells in a detectable tumor is very large ($M \sim 10^9$). Therefore, we concentrate on results that are asymptotically exact under the following limits:

$$u \to 0, \qquad M \to \infty, \qquad Mu^k = \text{constant}. \tag{1}$$

Above, k will be 1 or 2, depending on the result being presented. We will represent such a limit by the arrow $\xrightarrow[Mu^k=\text{const.}]{M\to\infty}$.

2 Number of resistant mutants at detection

We are first interested in following question: how many cells in the tumor are resistant to all D drugs at the time of detection?

2.1 Pathwise analysis

We begin by examining a single path, starting with fully sensitive cells (profile $0\ldots0$, indexed $j=0$), and ending with fully resistant cells (profile $1\ldots1$, indexed $j=m$). For each $j=0,\ldots,m$, we let $x_i(t)$ denote the expected number of cells of profile j at time t. These expected numbers satisfy the following system of differential equations:

$$\dot{x}_j = \big(r - b(\nu_{j+1}+\eta_{j+1})u\big)x_j + b\nu_j u x_{j-1}. \tag{2}$$

Above, we set $x_{-1}(t)=0$ for all t and $\nu_0=\eta_0=\nu_{m+1}=\eta_{m+1}=0$. The initial conditions are $x_0(0)=1$, and $x_j(0)=0$ for $j=1,\ldots,m$.

We assume for the moment that $\nu_k+\eta_k \neq \nu_\ell+\eta_\ell$ for all pairs $k,\ell = 1,\ldots,m$ with $k\neq\ell$. Under this assumption, the solution to the system (2)

is given by

$$x_j(t) = \begin{cases} e^{(r-b(\nu_1+\eta_1)u)t} & j=0 \\ e^{rt}\left(\prod_{k=1}^{j}\nu_k\right)\sum_{k=1}^{j+1}\frac{e^{-b(\nu_k+\eta_k)ut}}{\prod\limits_{\substack{1\le\ell\le j+1\\ \ell\neq k}}(\nu_\ell+\eta_\ell-\nu_k-\eta_k)} & 1\le j\le m-1 \\ e^{rt}\left(\prod_{k=1}^{m}\nu_k\right)\left(\frac{1}{\prod_{\ell=1}^{m}(\nu_\ell+\eta_\ell)}\right. & \\ \qquad \left. -\sum_{k=1}^{m}\frac{e^{-b(\nu_k+\eta_k)ut}}{(\nu_k+\eta_k)\prod\limits_{\substack{1\le\ell\le m\\ \ell\neq k}}(\nu_\ell+\eta_\ell-\nu_k-\eta_k)}\right) & j=m. \end{cases} \tag{3}$$

We note that the above expressions for $x_j(t)$ are expectations over all possible trajectories, including those in which the tumor becomes extinct. Since we are only interested in tumors that grow to detectable size, we divide these expressions by the tumor survival probability, which is r/b. This yields an exact expression for the expected number of cells of each type at time t, conditioned on the survival of the tumor.

However, we wish to know the expected number of resistant cells not at a fixed time from when the tumor was initiated, but at the moment the total number of cells reaches M. The time T for the tumor to reach M cells is a random quantity; however, we can approximate T by using the deterministic growth law $x(t) = b/r\, e^{rt}$ for the total population of cells. (In other words, we set the total cell population equal to its expected value conditioned on non-extinction.) We then solve $x(T) = M$, yielding the approximation

$$T \approx \frac{1}{r}\log(Mr/b). \tag{4}$$

Simulation results (not shown) suggest that this approximation is exact in the large tumor size, small mutation rate limit (1).

We approximate the expected number x_m^{det} of fully resistant cells at detection (arising via this particular path) by substituting approximation (4)

for T into the expression for $x_m(t)$ given in (3). This yields

$$x_m^{\text{det}} = M\left(\prod_{k=1}^m \nu_k\right)\left(\frac{1}{\prod_{\ell=1}^m(\nu_\ell+\eta_\ell)} - \sum_{k=1}^m \frac{e^{-\frac{b}{r}(\nu_k+\eta_k)u\log(Mr/b)}}{(\nu_k+\eta_k)\prod_{\substack{1\le\ell\le m\\ \ell\neq k}}(\nu_\ell+\eta_\ell-\nu_k-\eta_k)}\right). \tag{5}$$

To simplify the above expression, we define $\mu = (b/r)\,u\log(Mr/b)$. We note that $\mu \to 0$ in the rare mutation, large tumor size limit $u \to 0$, $M \to \infty$, Mu = constant. Substituting μ into (5) and replacing $e^{-(\nu_k+\eta_k)\mu}$ by its Taylor expansion, we obtain

$$\begin{aligned} x_m^{\text{det}} &= M\left(\prod_{k=1}^m \nu_k\right)\left(\frac{1}{\prod_{\ell=1}^m(\nu_\ell+\eta_\ell)} - \sum_{k=1}^m \frac{\sum_{s=0}^{\infty}\frac{\mu^s}{s!}(-\nu_k-\eta_k)^s}{(\nu_k+\eta_k)\prod_{\substack{1\le\ell\le m\\ \ell\neq k}}(\nu_\ell+\eta_\ell-\nu_k-\eta_k)}\right) \\ &= M\left(\prod_{k=1}^m \nu_k\right) \\ &\quad \left(\frac{1}{\prod_{\ell=1}^m(\nu_\ell+\eta_\ell)} - \sum_{s=0}^{\infty}\frac{\mu^s}{s!}\sum_{k=1}^m \frac{(-\nu_k-\eta_k)^s}{(\nu_k+\eta_k)\prod_{\substack{1\le\ell\le m\\ \ell\neq k}}(\nu_\ell+\eta_\ell-\nu_k-\eta_k)}\right). \end{aligned} \tag{6}$$

For any collection of m distinct nonzero real numbers $\alpha_1,\ldots,\alpha_m$—in our case, we are interested in $\alpha_j = \nu_j + \eta_j$—the following combinatorial identity holds:

$$\sum_{j=1}^m \frac{(-\alpha_j)^s}{\alpha_j\prod_{\substack{1\le\ell\le m\\ \ell\neq j}}(\alpha_\ell-\alpha_j)} = \begin{cases} \frac{1}{\prod_{j=1}^m \alpha_j} & s=0 \\ 0 & 1\le s\le m-1 \\ -1 & s=m. \end{cases} \tag{7}$$

We save the proof of this identity for Section 7. Using this identity to simplify (6), we obtain

$$x_m^{\text{det}} = M\left(\prod_{j=1}^m \nu_j\right)\frac{\mu^m}{m!} + \mathcal{O}(\mu^{m+1}). \tag{8}$$

Interestingly, this formula does not involve the numbers η_j of off-path mutations. As a consequence, we see that this result does not depend on the assumption that $\nu_k + \eta_k \neq \nu_\ell + \eta_\ell$ for $k \neq \ell$, and holds regardless of whether this condition is satisfied.

2.2 One drug

For single-drug therapy ($D = 1$), there is only one path to resistance, and the expected number of resistant cells at detection is given by (5), which simplifies to

$$x_{\text{res}}^{\text{det}} = M(1 - e^{-n_1\mu}) = Mn_1\mu + \mathcal{O}(\mu^2). \tag{9}$$

(We recall from above that $\mu = (b/r)\, u \log(Mr/b)$.)

2.3 Two drugs

For two-drug therapy, there are three paths to consider:

Path 1: $00 \to 10 \to 11$ In this case we have $\nu_1 = n_1$ and $\nu_2 = n_2 + n_{12}$. Using (8) we obtain

$$x_{\text{res},00\to10\to11}^{\text{det}} \approx Mn_1(n_2 + n_{12})\frac{\mu^2}{2} + \mathcal{O}(\mu^3).$$

Path 2: $00 \to 01 \to 11$ Similarly to path 1, we obtain

$$x_{\text{res},00\to01\to11}^{\text{det}} \approx Mn_2(n_1 + n_{12})\frac{\mu^2}{2} + \mathcal{O}(\mu^3).$$

Path 3: $00 \to 11$ Here $\nu_1 = n_{12}$. Using the exact solution (5) we obtain

$$x_{\text{res},00\to11}^{\text{det}} = M(1 - e^{-n_{12}\mu}) \approx M\left(n_{12}\mu - n_{12}^2\frac{\mu^2}{2}\right) + \mathcal{O}(\mu^3).$$

Above, we have taken a second-order Taylor expansion of $e^{-n_{12}\mu}$.

Aggregating the above results, we obtain the following expression for the total expected number of resistant cells at detection:

$$x_{\text{res}}^{\text{det}} = M\left\{n_{12}\mu + \left[2n_1n_2 + n_{12}(n_1 + n_2 - n_{12})\right]\frac{\mu^2}{2}\right\} + \mathcal{O}(\mu^3). \tag{10}$$

2.4 Arbitrary number of drugs with no cross-resistance

Suppose a therapy consists of $D \geq 1$ drugs, and there are no mutations that simultaneously confer resistance to multiple drugs. In this case, using (8), we obtain a remarkably simple formula for the expected number of resistant cells at detection:

$$x_{\text{res}}^{\text{det}} = Mn_1 \cdots n_D \mu^D + \mathcal{O}(\mu^{D+1}). \quad (11)$$

For example, in the case of triple therapy with no cross-resistance, we expect approximately $Mn_1n_2n_3\mu^3$ resistant cells at detection (accurate to order μ^4). Notice that the factorial in (8) is cancelled by the number, $D!$, of possible paths to full resistance.

3 Generating functions for branching processes

Generating functions are a powerful tool for analyzing stochastic processes. In a generating function, the probabilities of different events are recorded as coefficients in a power series, allowing these probabilities to be easily manipulated. Here we introduce the generating functions for the one- and two-type branching processes, which we will later use to derive probabilities of resistance and of treatment success.

3.1 One-type branching process

We first consider the one-type branching process. We introduce the random variable $Y(t)$ to represent the number of cells at time t, given that there was one such cell at time $t = 0$. The generating function for this process is then defined as

$$\phi_{m=1}(z;t) \equiv \mathrm{E}\left[z^{Y(t)}\right] \equiv \sum_{y=0}^{\infty} \mathrm{P}[Y(t) = y]\, z^y.$$

In words, the generating function is a time-dependent power series in which the coefficient of z^y equals the probability that there were y cells at time t. There is a well-known closed-form expression for this generating function (Athreya and Ney, 2004):

$$\phi_{m=1}(z;t) = \frac{d(1-z) + (zb-d)e^{-rt}}{b(1-z) + (zb-d)e^{-rt}}.$$

In particular, the probability that a lineage of type 1 cells survives for time t is given by

$$1 - \phi_{m=1}(0;t) = \frac{r}{b - de^{-rt}}. \quad (12)$$

3.2 Two-type branching process

We now turn to a branching process involving two types, labeled 1 and 2, with one-way mutation of rate $u\nu_2$ from type 1 to type 2. This process can be described by the following rates:

$$\begin{cases} 1 \to 11 & \text{rate } b \\ 1 \to \emptyset & \text{rate } d \\ 1 \to 12 & \text{rate } bu\nu_2 \\ 2 \to 22 & \text{rate } b \\ 2 \to \emptyset & \text{rate } d. \end{cases} \tag{13}$$

We introduce the random variables $Y_1(t)$ and $Y_2(t)$ to represent the numbers of type 1 and type 2 cells at time t, given that the process was initiated with a single type 1 cell at time $t = 0$. The generating function for this process is defined as

$$\phi_{m=2}(z_1, z_2; t) \equiv \mathrm{E}\left[z_1^{Y_1(t)} z_2^{Y_2(t)}\right] \equiv \sum_{y_1, y_2 \geq 0} \mathrm{P}\left[Y_1(t) = y_1, Y_2(t) = y_2\right] z_1^{y_1} z_2^{y_2}.$$

In words, the generating function is a time-dependent power series in the variables z_1 and z_2, with the coefficient of $z_1^{y_1} z_2^{y_2}$ equal to the probability that there are y_1 cells of type 1 and y_2 cells of type 2 at time t.

A closed-form expression for this branching process was discovered by Antal and Krapivsky (2011). To state this solution, we first define the following constants:

$$\alpha = \frac{1}{2}\left[-(1 - u\nu_2) + \sqrt{(1 - u\nu_2)^2 + 4u\nu_2 \frac{b}{r}}\right],$$

$$\beta = 1 + \sqrt{(1 - u\nu_2)^2 + 4u\nu_2 \frac{b}{r}}.$$

Next, we let F denote the hypergeometric function ${}_2F_1$, and we define the following functions of a real number x:

$$F_1(x) = F(\alpha, 1 + \alpha, \beta; x),$$
$$F_2(x) = F(1 + \alpha - \beta, 2 + \alpha - \beta, 2 - \beta; x),$$
$$F_3(x) = \frac{\alpha(1 + \alpha)}{\beta} F(1 + \alpha, 2 + \alpha, 1 + \beta; x),$$
$$F_4(x) = \frac{(1 + \alpha - \beta)(2 + \alpha - \beta)}{2 - \beta} F(2 + \alpha - \beta, 3 + \alpha - \beta, 3 - \beta; x).$$

Third, we define the following quantities, which depend on the arguments z_1, z_2, and t, of the generating function $\phi_{m=2}(z_1, z_2; t)$:

$$y_0 = 1 - \frac{r}{b(1-z_2)},$$
$$\kappa = \frac{1}{y_0}\left[\frac{b}{r}(z_1 - 1) - \alpha\right],$$
$$y = y_0 e^{-rt},$$
$$C = y_0^{\beta} \frac{\kappa F_1(y_0) - F_3(y_0)}{(1-\beta-\kappa y_0)F_2(y_0) + y_0 F_4(y_0)}.$$

Finally, we state Antal and Krapivsky's (2011) formula for the generating function of the two-type branching process in terms of the above quantities and functions:

$$\phi_{m=2}(z_1, z_2; t) = 1 + \frac{r}{b}\alpha + \frac{r}{b}\frac{y^{\beta}F_3(y) + C(1-\beta)F_2(y) + CyF_4(y)}{y^{\beta-1}F_1(y) + CF_2(y)}. \quad (14)$$

4 Probability of resistance at time of detection

We now turn to the question of whether at least one resistant cell exists at the time the tumor reaches detectable size. Again we consider each path to resistance separately.

4.1 One-step paths

A number of works (Coldman and Goldie, 1983; Dewanji et al., 2005; Komarova and Wodarz, 2005; Iwasa et al., 2006) have investigated the probability that resistance exists at the start of treatment, in the case that this resistance can be achieved through a single mutation. Here we follow the approach of Dewanji et al. (2005), in which we suppose that type 0 (sensitive) cells grow deterministically, and that type 1 (resistant) cells arise as a Poisson process, with rate depending on the current number of type 0 cells. Specifically, we approximate the growth of type 0 cells (conditioned on non-extinction) by

$$x_0(t) = \frac{b}{r}e^{rt}. \quad (15)$$

Noting that type 0 cells divide at rate b, and each division produces a type 1 mutant with probability $u\nu_1$, we suppose that type 1 (resistant) cells arise as a Poisson process with rate $bu\nu_1 x_0(t)$ at time t. Each line of type 1 cells

thus created is described by a one-type branching process initiated at the time of mutation.

We let the random variable $X_1(t)$ denote the number of type 1 cells at time t. The probability that there are no resistant cells at the time of detection ($T = 1/r \log(Mr/b)$) is equal to the probability that none of the type 1 mutations that arise in the interval $[0, T]$ survive to time T. The probability that a single type 1 mutation arising at time $0 \leq s \leq T$ survives until time T is given by $1 - \phi_{m=1}(0; T - s)$. Recalling that these mutations arise at rate $bu\nu_1 x_0(s)$ for $0 \leq s \leq T$, we can write this probability as

$$\mathrm{P}\left[X_1(T) = 0\right] = \exp\left\{-\int_0^T bu\nu_1 x_0(s)\left[1 - \phi_{m=1}(0; T - s)\right] ds\right\}.$$

Substituting from (12) and simplifying, we arrive at

$$\begin{aligned}\mathrm{P}[X_1(T) = 0] &= \exp\left\{-Mu\nu_1 \frac{b}{d} \log\left[\frac{b}{r}\left(1 - \frac{d}{rM}\right)\right]\right\} \\ &\xrightarrow[Mu=\text{const.}]{M \to \infty} \exp\left[-Mu\nu_1 \frac{b}{d} \log\left(\frac{b}{r}\right)\right].\end{aligned} \tag{16}$$

This result was also obtained by Iwasa et al. (2006).

4.2 Two-step paths

We can apply similar methods to two-step paths ($m = 2$). The probability that, time t after a type 1 cell arises, this cell's lineage includes at least one surviving type 2 cell, can be written as $1 - \phi_{m=2}(1, 0, t)$. Thus a type 1 mutation that arises at time s, $0 \leq s \leq T$, will give rise to at least one living type 2 cell at time T, with probability $1 - \phi_{m=2}(1, 0, T - s)$. Following the arguments in the $m = 1$ case, the probability of no type 2 cells at time T can be written as

$$\mathrm{P}\left[X_2(T) = 0\right] = \exp\left\{-\int_0^T bu\nu_1 x_0(s)\left[1 - \phi_{m=2}(1, 0; T - s)\right] ds\right\}. \tag{17}$$

4.3 Low-mutation expansion for two-step paths

For low mutation rates, we can expand (17) as follows. First, we work through the construction of $\phi_{m=2}(z_1, z_2; t)$, substituting $z_1 = 1$ and $z_2 = 0$,

and expanding all quantities and functions to low orders in u. This yields:

$$\begin{aligned}
\alpha &= \frac{b}{r} u\nu_2 + \mathcal{O}(u^2), \\
\beta &= 2 + \left(2\frac{b}{r} - 1\right) u\nu_2 + \mathcal{O}(u^2), \\
y_0 &= 1 - \frac{r}{b}, \\
\kappa &= -\frac{\alpha}{y_0} = -u\nu_2 \frac{b^2}{r(b-r)} + \mathcal{O}(u^2),
\end{aligned}$$

$$\begin{aligned}
F_1(x) &= 1 + \mathcal{O}(u), \\
F_2(x) &= 1 - x\frac{b/r - 1}{2b/r - 1} + \mathcal{O}(u), \\
F_3(x) &= -\frac{b}{r} u\nu_2 \frac{1}{x}\left[1 + \frac{1}{x}\log(1-x)\right] + \mathcal{O}(u^2), \\
F_4(x) &= -\frac{b/r - 1}{2b/r - 1} + \mathcal{O}(u), \\
C &= u\nu_2 \frac{b}{r} \log\left(\frac{b}{r}\right) + \mathcal{O}(u^2), \\
y(t) &= \left(1 - \frac{r}{b}\right) e^{-rt}.
\end{aligned}$$

Combining the above expansions yields the following expansion for $\phi_{m=2}(1, 0; t) - 1$:

$$\phi_{m=2}(1, 0; t) - 1 = -u\nu_2 \frac{\log\left[\frac{b}{r} - \left(\frac{b}{r} - 1\right) e^{-rt}\right]}{\left(1 - \frac{r}{b}\right) e^{-rt}} + \mathcal{O}(u^2). \tag{18}$$

Substituting (18) into formula (17) for the probability of no resistance yields

$$\begin{aligned}
&\mathrm{P}\left[X_2(T) = 0\right] \\
&= \exp\left\{-Mu^2\nu_1\nu_2 \frac{b^2}{b-r} \int_0^T \log\left[\frac{b}{r} - \left(\frac{b}{r} - 1\right) e^{-r(T-s)}\right] ds\right\} + \mathcal{O}(u^3)
\end{aligned} \tag{19}$$

Above, we have also used the substitution $M = b/r\, e^{rT}$. We simplify the

integral in (19) as follows:

$$\begin{aligned}
&\int_0^T \log\left[\frac{b}{r} - \left(\frac{b}{r} - 1\right) e^{-r(T-s)}\right] ds \\
&= \int_0^T \log\left[\frac{b}{r} - \left(\frac{b}{r} - 1\right) e^{-rt}\right] dt \\
&= \log\left(\frac{b}{r}\right) T + \int_0^T \log\left[1 - \left(1 - \frac{r}{b}\right) e^{-rt}\right] dt.
\end{aligned}$$

The remaining integral above is positive and bounded above by

$$\int_0^\infty \log\left[1 - \left(1 - \frac{r}{b}\right) e^{-rt}\right] dt = \frac{1}{r} \operatorname{Li}_2\left(1 - \frac{r}{b}\right),$$

where Li_2 is the dilogarithm function. In particular, this bound is constant with respect to T, and therefore becomes negligible in comparison to $\log(b/r)T$ as T becomes large. This implies the following asymptotic formula for the integral in (19):

$$\int_0^T \log\left[\frac{b}{r} - \left(\frac{b}{r} - 1\right) e^{-r(T-s)}\right] ds \xrightarrow[T\to\infty]{} \log\left(\frac{b}{r}\right) T.$$

Finally, substituting the above result into (19), and additionally substituting $T = \log(Mr/b)/r$, we obtain

$$\mathrm{P}\left[X_2(T) = 0\right] \approx \exp\left[-Mu^2\nu_1\nu_2 \frac{b^2}{r(b-r)} \log\left(\frac{b}{r}\right) \log\left(\frac{Mr}{b}\right)\right]. \tag{20}$$

Our formulas (17) and (20) improve on results obtained by Haeno et al. (2007), as we discuss in Section 6.2.

4.4 Overall probability of resistance

One drug For a single drug, the probability that resistance exists at the time of detection can be obtained directly from (16) with $\nu_1 = n_1$, yielding

$$\begin{aligned}
p_{\text{res}} &= 1 - \exp\left\{-Mun_1 \frac{b}{d} \log\left[\frac{b}{r}\left(1 - \frac{d}{rM}\right)\right]\right\} \\
&\xrightarrow[Mu=\text{const.}]{M\to\infty} \quad 1 - \exp\left[-Mun_1 \frac{b}{d} \log\left(\frac{b}{r}\right)\right].
\end{aligned}$$

Two drugs For two drugs, we must consider the one-step path $00 \to 11$ and the two two-step paths $00 \to 10 \to 11$ and $00 \to 01 \to 11$. The probability that resistance exists at the time of detection equals one minus the probability that no cells of profile 11 are generated via any of these paths. We write this probability as $p_{\text{res}} = 1 - p_1 p_2$, where

$$p_1 = \exp\left[-Mun_{12}\frac{b}{d}\log\left(\frac{b}{r}\right)\right]$$

is obtained from (16) with $\nu_1 = n_{12}$, and

$$p_2 \approx \exp\left[-Mu^2\big(2n_1n_2 + n_{12}(n_1+n_2)\big)\frac{b^2}{r(b-r)}\log\left(\frac{b}{r}\right)\log\left(\frac{Mr}{b}\right)\right]$$

is obtained from (20) with $\nu_1 = n_1$, $\nu_2 = n_2 + n_{12}$ for the path $00 \to 10 \to 11$, and $\nu_1 = n_2$, $\nu_2 = n_1 + n_{12}$ for the path $00 \to 01 \to 11$. Rewriting in terms of $s = 1 - d/b$ yields the formulas for p_1 and p_2 presented in the main text.

5 Probability of tumor eradication

We now turn to the ultimate success or failure of multi-drug therapy. We define the therapy as successful if the tumor ultimately becomes extinct; otherwise, the tumor grows exponentially and there is a relapse. This differs from the question of analyzed in Section 4—the probability that resistance is present at the start of treatment—for two reasons. First, resistance that exists at the start of treatment may disappear due to stochastic drift. Second, new resistance mutations may appear during therapy.

We recall that, for all cell types sensitive to at least one drug (that is, all cells with resistance profiles other than $11\ldots1$), the birth and death rates during treatment are denoted b' and d', respectively, with $r' = b' - d' < 0$. Cells with resistance to all drugs are unaffected.

We separate the question of ultimate treatment outcome into resistance arising during tumor expansion and resistance arising during treatment. We let $p^{\uparrow}$ and $p^{\downarrow}$ denote the probability that no resistance mutations leading to relapse arise during expansion and treatment, respectively. The overall probability of eradication can then be written

$$p_{\text{erad}} = p^{\uparrow}p^{\downarrow}.$$

5.1 Resistance arising during expansion

We further separate into paths leading toward resistance.

5.1.1 One-step paths

As in Section 4.1, we suppose that type 1 (resistant) cells arise as a Poisson process with rate $bu\nu_1 x_0(t)$ at time t, with $x_0(t) = b/r\, e^{rt}$. We also recall that each type 1 (resistant) mutation that arises has probability r/b of ultimately escaping stochastic drift (leading to unchecked tumor growth and patient relapse). Using similar reasoning to Section 4.1, the probability that no resistance mutations leading to treatment failure arise by this path, during tumor expansion, can be written as

$$\begin{aligned} p^{\uparrow}_{m=1} &= \exp\left[-\int_0^T bu\nu_1 x_0(s)\,\frac{r}{b}\,ds\right] \\ &= \exp\left[-bu\nu_1\int_0^T e^{rs}\,ds\right] \\ &= \exp\left[-u\nu_1(M - b/r)\right] \\ &\xrightarrow[Mu=\text{const.}]{M\to\infty} e^{-Mu\nu_1}. \end{aligned} \tag{21}$$

This result was previously obtained by Komarova (2006, Appendix A).

5.1.2 Two-step paths

During the treatment phase, the lineage of each type 2 (fully resistant) cell will ultimately disappear with probability $\chi_2 = d/b$. For a type 1 cell that is present at the start of treatment, the probability χ_1 that its lineage will ultimately disappear can be obtained from the results of Antal and Krapivsky (2011):

$$\begin{aligned} \chi_1 &= \frac{1}{2}\left(1 + \frac{d'}{b'} + u\nu_2\frac{r}{b} - \sqrt{\left(1 + \frac{d'}{b'} + u\nu_2\frac{r}{b}\right)^2 - 4\frac{d'}{b'}}\right) \\ &= 1 + u\nu_2\frac{r}{b}\frac{b'}{r'} + \mathcal{O}(u^2). \end{aligned} \tag{22}$$

Above, b' and d' are the birth and death rates, respectively, of type 1 cells during treatment, and $r' = b' - d'$.

Suppose a type 1 mutation gives rise to y_1 type 1 cells and y_2 type 2 cells at the time of detection. Then the probability that all lineages of these cells disappear during treatment (and thus none of them cause eventual relapse) is $\chi_1^{y_1}\chi_2^{y_2}$. Overall, for a type 1 mutation that arises at time s, $0 \le s \le T$,

the probability that the lineage of this cell eventually disappears can be written as

$$\sum_{y_1,y_2\geq 0} \mathrm{P}\left[Y_1(T-s)=y_1, Y_2(T-s)=y_2\right]\chi_1^{y_1}\chi_2^{y_2} \quad = \quad \phi_{m=2}(\chi_1,\chi_2,T-s).$$

Following the arguments of previous sections, the probability that no type 1 mutation leading to treatment failure arises during expansion can be written as

$$p^{\uparrow}_{m=2} = \exp\left\{-\int_0^T bu\nu_1 x_0(s)\big[1-\phi_{m=2}(\chi_1,\chi_2;T-s)\big]\, ds\right\}. \tag{23}$$

This formula involves the expression $\phi_{m=2}(\chi_1,\chi_2;T-s)$. This expression cannot be evaluated directly using formula (14) for $\phi_{m=2}$, obtained by Antal and Krapivsky (2011), because this formula is undefined (has a removable singularity) at $z_2=\chi_2=d/b$. We therefore derive an expression for $\phi_{m=2}(z_1,d/b;t)$ from first principles. To begin, we note that dynamics of the two-type branching process (13) satisfy the backward Kolmogorov equations, which can be written as:

$$\begin{aligned}\partial_t\phi &= b\phi^2 + d + b\nu_2 u\phi\,\tilde{\phi} - (b+d+b\nu_2 u)\phi \\ \partial_t\tilde{\phi} &= b\tilde{\phi}^2 + d - (b+d)\tilde{\phi}.\end{aligned} \tag{24}$$

Above, $\phi\equiv\phi_{m=2}(z_1,z_2;t)$ is the generating function for the two-type process starting with a cell of type 1, while $\tilde{\phi}(z_1,z_2;t)$ is the analogous generating function starting with a cell of type 2. From the second equation in (24), we can see that

$$\tilde{\phi}(z_1,d/b;t)=d/b,$$

for all z_1 and all $t>0$. Thus, for the fixed value $z_2=\chi_2=d/b$, the backward Kolmogorov equations (24) reduce to

$$\partial_t\phi = b\phi^2 - (b+d+r\nu_2 u)\phi + d.$$

This is a Ricatti equation with constant coefficients, which can be solved using standard techniques. We find the solution

$$\phi_{m=2}(z_1,\chi_2;t) = 1 - \frac{\dfrac{\theta_+ e^{b\theta_+ t}}{1-z_1-\theta_+} - \dfrac{\theta_- e^{b\theta_- t}}{1-z_1-\theta_-}}{\dfrac{e^{b\theta_+ t}}{1-z_1-\theta_+} - \dfrac{e^{b\theta_- t}}{1-z_1-\theta_-}}. \tag{25}$$

Above,

$$\theta_\pm = \frac{1}{2}\left[\frac{r}{b}(1-u\nu_2) \pm \sqrt{\left(\frac{r}{b}(1-u\nu_2)\right)^2 + 4u\nu_2\frac{r}{b}}\right]. \tag{26}$$

5.1.3 Low-mutation expansion for two-step paths

We now derive an expression for $p^{\uparrow}_{m=2}$, the probability that no resistance arises via the path in question during tumor expansion, that is asymptotically exact in the limit of small mutation rate u and large detection size M. We know $\chi_2 = d/b$, and from (22) we have

$$\chi_1 = 1 + u\nu_2 \frac{r}{b}\frac{b'}{r'} + \mathcal{O}(u^2).$$

The formula (26) for $\theta_\pm$ admits the following low-mutation expansions:

$$\theta_+ = \frac{r}{b} + O(u), \qquad \theta_- = -u\nu_2 + O(u^2).$$

We substitute these expansions into (25) and note that, for values of t that contribute significantly to the integral in (23) (with t identified as $T-s$), the terms containing e^{rt} eclipse those that are constant in t. As $u \to 0$ and $e^{rt} \to \infty$, we have

$$1 - \phi(\chi_1, \chi_2, t) \to \frac{r}{b}\left(1 + \frac{1}{u\nu_2 a}e^{-rt}\right)^{-1}, \qquad a = \frac{b}{r} - \frac{b'}{r'}. \tag{27}$$

To obtain $p^{\uparrow}_{m=2}$ we substitute (27) into (23), which yields

$$p^{\uparrow}_{m=2} \xrightarrow[Mu^2=\text{const.}]{M\to\infty} \exp\left(-bu\nu_1 \int_0^T \frac{1}{e^{-rs}+A}ds\right), \qquad A = (au\nu_2 e^{rT})^{-1}. \tag{28}$$

The integral on the right-hand side above simplifies as follows:

$$\int_0^T \frac{1}{e^{-rs}+A}ds = \frac{1}{Ar}\log\frac{1+Ae^{rT}}{1+A} \xrightarrow{e^{rT}\to\infty} \frac{1}{Ar}\log\frac{Ae^{rT}}{1+A}.$$

Finally, by expressing everything in terms of the tumor size at detection, $M = b/r\, e^{rT}$, we arrive at

$$p^{\uparrow}_{m=2} \xrightarrow[Mu^2=\text{const.}]{M\to\infty} \exp\left[Mu^2\nu_1\nu_2 a \log\left(\frac{b}{rM} + u\nu_2 a\right)\right], \qquad a = \frac{b}{r} - \frac{b'}{r'}. \tag{29}$$

5.2 Resistance arising during treatment

5.2.1 One-step paths

For one-step paths, the probability that no resistance arises during treatment was obtained by Michor et al. (2006):

$$p^{\downarrow}_{m=1} = \exp\left(Mu\nu_1 \frac{r}{b}\frac{b'}{r'}\right). \tag{30}$$

5.2.2 Two-step paths

During treatment, type 1 mutations arise from type 0 cells at rate $b'u\nu_1 x_0(t)$ per unit time. Each such type 1 mutation has probability $1-\chi_1$ of avoiding disappearance due to drift and eventually causing relapse, where χ_1 is the extinction probability given by (22). Thus the probability that no type 1 mutations leading to relapse arise during treatment is given by

$$\begin{aligned} p^{\downarrow}_{m=2} &= \exp\left[-b'u\nu_1 \int_0^\infty M e^{r's}(1-\chi_1)\, ds\right] \\ &= \exp\left[\frac{b'}{r'}u\nu_1 M(1-\chi_1)\right] \\ &\xrightarrow[Mu^2=\text{const.}]{M\to\infty} \exp\left[-Mu^2\nu_1\nu_2\frac{r}{b}\left(\frac{b'}{r'}\right)^2\right]. \end{aligned} \tag{31}$$

5.3 Overall probability of tumor eradication

One drug For single-drug therapy, the probability of eradication is obtained by combining (21) and (30):

$$p_{\text{erad}} = p^{\uparrow}_{0\to 1}p^{\downarrow}_{0\to 1} = \exp\left(-Mun_1\frac{r}{b}a\right), \qquad a = \frac{b}{r} - \frac{b'}{r'}.$$

Two drugs The probability of eradication for two-drug combination therapy can be written as $p_{\text{erad}} = p^{\uparrow}_1 p^{\downarrow}_1 p^{\uparrow}_2 p^{\downarrow}_2$, where the subscript 1 refers to the path $00 \to 11$ and the subscript 2 refers to the paths $00 \to 10 \to 11$ and $00 \to 01 \to 11$. From (21) and (30) respectively, with $\nu_1 = n_{12}$, we have

$$p^{\uparrow}_1 = \exp(-Mun_{12}),$$

and

$$p^{\downarrow}_1 = \exp\left(-Mun_{12}\frac{r}{b}\frac{b'}{r'}\right).$$

From (30), and (31) repsectively, with $\nu_1 = n_1$, $\nu_2 = n_2 + n_{12}$ for the path $00 \to 10 \to 11$, and $\nu_1 = n_2$, $\nu_2 = n_1 + n_{12}$ for the path $00 \to 01 \to 11$, we have

$$\begin{aligned} p^{\uparrow}_2 = \exp\Bigg\{Mu^2 a\Bigg[&n_1(n_2+n_{12})\log\left(\frac{b}{rM} + u(n_2+n_{12})a\right) \\ &+ n_2(n_1+n_{12})\log\left(\frac{b}{rM} + u(n_1+n_{12})a\right)\Bigg]\Bigg\}, \end{aligned}$$

and

$$p_2^{\downarrow} = \exp\left[-Mu^2\big(2n_1n_2 + n_{12}(n_1+n_2)\big)\frac{r}{b}\left(\frac{b'}{r'}\right)^2 \right],$$

with $a = b/r - b'/r'$ as above. Substituting $r/b = s$ and $r'/b' = s'$ yields the formulas presented in the main text. We can also combine the above expressions to obtain

$$\begin{aligned} p_{\text{erad}} = \exp\Bigg\{ &- Mun_{12}\frac{r}{b}a \\ &+ Mu^2 n_1(n_2+n_{12})\left[\frac{r}{b}\left(\frac{b'}{r'}\right)^2 + a\log\left(\frac{b}{rM} + u(n_2+n_{12})a\right)\right] \\ &+ Mu^2 n_2(n_1+n_{12})\left[\frac{r}{b}\left(\frac{b'}{r'}\right)^2 + a\log\left(\frac{b}{rM} + u(n_1+n_{12})a\right)\right]\Bigg\}. \end{aligned} \quad (32)$$

Formula (32) improves on prior results of Komarova (2006), as we discuss in Section 6.3.

6 Comparison to previous results

Aspects of the dynamics of combination cancer therapy and resistance have been investigated in a number of previous works—notably Komarova and Wodarz (2005), Komarova (2006), and Haeno et al. (2007). Our results improve on the results previously obtained in these works and provide a closer match to simulations. This improvement is due in part to our use of recent advances in the theory of branching processes (Antal and Krapivsky, 2011), which allow us to obtain results that are asymptotically exact in the rare-mutation, large-tumor-size limit.

6.1 Expected number of resistant cells at detection

The question of the expected number of resistant cells in a tumor of detectable size has also been investigated by Iwasa et al. (2006), in the case of one drug, and Haeno et al. (2007), in the case of two drugs (with no mutations conferring resistance to both simultaneously).

For one drug, in the case that resistant cells have the same division and death rates as sensitive cells in the absence of treatment, formula (10) of Iwasa et al. (2006) gives the following expression for the expected number

of resistant cells:

$$x_{\rm res}^{\rm det} \approx M\frac{b}{r}n_1 u \log(M). \tag{33}$$

Comparing to our result (9), we see that Iwasa et al.'s coincides with ours (to first order in u) except that $\log(Mr/b)$ in (9) is replaced by $\log(M)$ in (33). This discrepancy arises from the fact that Iwasa et al. assume the sensitive cell population grows deterministically as e^{rt}, and do not condition on survival of the tumor. We compare Iwasa et al.'s formula (33) and ours (9) to simulation results in Table S1.

In the case of two drugs, Haeno et al. (2007) derive the following expression for the average number resistant cells:

$$x_{\rm res}^{\rm det} \approx 2\left(\frac{b}{r}\right)^2 n_1 n_2 u^2 \sum_{x=1}^{M-1} \frac{M}{x}\log\frac{M}{x}. \tag{34}$$

Upon applying the approximation

$$\sum_{x=1}^{M-1} \frac{M}{x}\log\frac{M}{x} \quad \approx \quad \int_1^M \frac{M}{x}\log\frac{M}{x}\,dx \quad = \quad \frac{1}{2}M\log(M)^2,$$

formula (34) becomes

$$x_{\rm res}^{\rm det} \approx \left(\frac{b}{r}\right)^2 n_1 n_2 u^2 \log(M)^2.$$

As in the result of Iwasa et al. (2006), this expression differs from ours only in the replacement of $\log(Mr/b)$ by $\log(M)$, which again arises because Haeno et al. do not condition on survival of the tumor. We compare this expression to our formula (10) in Table S2.

For three or more drugs (or for drug resistance requiring three or more mutational steps) our formulas (5), (8), and (11) provide the first closed-form expressions for the expected number of fully resistant cells at time of detection.

6.2 Probability of resistance at detection

Haeno et al. (2007) also derive expressions for $\mathrm{P}\left[X_2(T) > 0\right]$, the probability that resistance requiring two mutational steps is present at the time of detection. Their expressions are derived using approximate solutions to the differential equations that define the generating function $\phi_{m=2}(z_1, z_2; t)$

M	n_1	Simulation	This work, Eq. (9)	Iwasa et al., Eq. (33)
10^5	1	53	50	58
10^5	10	510	494	575
10^5	100	4900	4831	5754
10^7	1	76	73	81
10^7	10	740	725	806
10^7	100	7400	7252	8059
10^9	1	96	96	104
10^9	10	920	956	1036
10^9	100	9800	9557	10361

Table S1: Comparison of formulas and simulation results for the expected number of resistant cells at detection for the case of one drug. Parameter values are $b = 0.25$, $d = 0.2$, and $u = 1/M$ for each value of M. 10^8 simulation runs were used per parameter combination.

M	n_1	n_2	Simulation	This work, Eq. (10)	Haeno et al., (34)
10^5	10	10	2.7	2.5	3.6
10^5	100	100	250	245	365
10^5	1000	1000	14000	24520*	36133
10^7	10	10	0.05	0.05	0.07
10^7	100	100	5.6	5.26	6.96
10^7	1000	1000	550	526	696
10^9	10	10	0.001	0.001	0.001
10^9	100	100	0.09	0.09	0.11
10^9	1000	1000	9.4	9.1	11.3

Table S2: Comparison of formulas and simulation results for the expected number of resistant cells at detection for the case of two drugs. Parameter values: $b = 0.25$, $d = 0.2$, $u = 1/M$, $n_{12} = 0$. 10^8 simulation runs were used per parameter combination. *The high degree of inaccuracy for this parameter combination occurs because $un_1 = un_2 = 10^{-2}$ is sufficiently large to introduce errors in approximations that assume $un_i \ll 1$.

M	Sim.	This work, Eq. (17) (exact)	This work, Eq. (20) (closed-form)	Haeno et al., Eq. (35)	Haeno et al., Eq. (36)
5×10^8	0.10	0.10	0.17	0.32	0.029
1×10^9	0.20	0.20	0.32	0.57	0.051
2×10^9	0.36	0.35	0.55	0.9	0.09
3×10^9	0.48	0.48	0.70	*	0.12
5×10^9	0.67	0.66	0.88	*	0.17
1×10^{10}	0.89	0.89	0.98	*	0.27

Table S3: Probability p_{res} of resistance at detection for dual therapy with no cross-resistance, as calculated using simulation, using our formulas (17) and (20), and using the formulas (35) and (36) of Haeno et al. (2007). Parameter values are $u = 10^{-8}$, $n_1 = n_2 = 100$, $b = b' = 0.25$, $d = 0.2$. *Here Haeno et. al's formula fails by giving a probability greater than 1.

(see Appendix A). In contrast, our formulas (17) and (20) for $\mathrm{P}\left[X_2(T) = 0\right]$ utilize the exact solution obtained by Antal and Krapivsky (2011).

The two formulas derived by Haeno et al. (2007) both describe the probability that, at the time of detection, at least one cell contains two mutations arising in a specified order. (In other words, their formulas apply to the case of a particular two-step path.) Their main formula can be expressed in our notation as

$$\mathrm{P}\left[X_2(T) > 0\right] = -\frac{Mu^2\nu_1\nu_2\log(u\nu_2)}{(1-d/b)^2}. \tag{35}$$

They also present an alternative formula:

$$\mathrm{P}\left[X_2(T) > 0\right] = \sum_{x=1}^{M} e^{-u\nu_1(x-1)}\left(1 - e^{-u\nu_1}\right)\left[1 - \exp\left(-\frac{Mu_2}{(1-d/b)(x+1)}\right)\right]. \tag{36}$$

In Table S3 we compare values of the overall probability of no resistance at detection p_{res} (along all paths) as obtained from simulation, as derived from our formulas (17) and (20), and as derived from formulas (35) and (36) of Haeno et al. (2007).

6.3 Probability of tumor eradication

Turning to the question of whether therapy will successfully eradicate a tumor, our results in the case of one-step paths to resistance agree with

those of Komarova (2006) and Michor et al. (2006), as noted where these results are presented.

For two-step paths, Komarova (2006) obtained closed-form approximations for the probability of tumor eradication, in the special case that an equal number of mutations are required for each step ($\nu_1 = \nu_2$), and that treatment affects only the death rate of tumor cells, not the division rate ($b' = b$).

Komarova's (2006) expression for the probability $p^{\uparrow}$—that no mutations leading to eventual relapse arise during tumor expansion—can be expressed in our notation as

$$p^{\uparrow} = 1 - M(u\nu)^2 \frac{b}{r} \log\left(\frac{M}{M_0} - 1\right). \tag{37}$$

Above, $\nu = \nu_1 = \nu_2$ is the number of resistance mutations at each step, and M_0 is an extra parameter representing tumor's initial size. Our approach of considering the entire history of the tumor, conditioned on its survival, amounts to setting $M_0 = b/r$. (We also note that Komarova uses $P^{\uparrow}$ and $P^{\downarrow}$ to denote the probabilities that mutations leading to relapse *do* arise during the two respective phases; thus $P^{\uparrow}$ in Komarova's notation corresponds to $1 - p^{\uparrow}$ in ours, and similarly for $P^{\downarrow}$ and $p^{\downarrow}$.)

The approximation (37) for $p^{\uparrow}$ is based on first determining the probability that at least one type 2 mutation arises, then multiplying that quantity by the survival probability of each such mutation. This method is accurate if few type 2 mutants are likely to be generated ($Mu^2\nu^2 \lesssim 1$), but loses accuracy if many type 2 mutations may arise, because it does not take into account the individual fate of each mutation. Our expression (29), based on the exact formula for $\phi_{m=2}(z_1, z_2; t)$ obtained by Antal and Krapivsky (2011), does not have this limitation.

For the probability $p^{\downarrow}$ that no mutations leading to relapse arising during treatment, Komarova (2006) obtained

$$p^{\downarrow} = \left[1 - (u\nu)^2 \frac{br}{(r')^2}\right]^M \approx \exp\left[-M(u\nu)^2 \frac{br}{(r')^2}\right]. \tag{38}$$

This expression coincides with our formula (31) in the special case $\nu_1 = \nu_2 = \nu$ and $b' = b$. Our result (31) can therefore be seen as a generalization of Komarova's result (38) to the case that mutation numbers may be unequal and treatment may affect tumor cell division rates.

Table S4 compares our formula (32) for the overall probability p_{erad} of tumor eradication for two-drug therapy to the results of Komarova (2006).

M	Simulation	This work, Eq. (32)	Komarova (2006)
10^9	0.79	0.79	0.80
2×10^9	0.63	0.62	0.59
3×10^9	0.50	0.49	0.38
4×10^9	0.39	0.38	0.17
5×10^9	0.31	0.30	*

Table S4: Probability p_{erad} of tumor eradication as calculated using simulation, using our formula (32), and using the formulas (37) and (38) of Komarova (2006). Parameter values are $u = 10^{-8}$, $n_1 = n_2 = 100$, $b = b' = 0.25$, $d = 0.2$, $d' = 0.3$. *Here Komarova's formula fails by giving a negative answer.

Finally, we note that Komarova (2006) also provides computational recipes to obtain probabilities for treatment success to arbitrary numerical precision. This methodology has been applied to a number of specific questions regarding treatment of chronic myeloid leukemia (Komarova and Wodarz, 2005; Komarova et al., 2009; Katouli and Komarova, 2010; Komarova, 2011).

7 Proof of Identity (7)

In this section we prove the mathematical identity used in Section 2.1. We state this identity in an equivalent form:

Theorem 1. *For any collection of m distinct nonzero real numbers $\alpha_1, \ldots, \alpha_m$ and any integer $0 \le s \le m$,*

$$\sum_{j=1}^{m} \frac{\alpha_j^{s-1}}{\prod\limits_{\substack{1 \le \ell \le m \\ \ell \neq j}} (\alpha_\ell - \alpha_j)} = \begin{cases} \frac{1}{\prod_{\ell=1}^{m} \alpha_\ell} & s = 0 \\ 0 & 1 \le s \le m-1 \\ (-1)^{m+1} & s = m. \end{cases} \tag{39}$$

Proof. Our proof is based on Cauchy's residue theorem of complex analysis. Consider the meromorphic function

$$F(z) = \frac{z^{s-1}}{\prod_{\ell=1}^{m} (\alpha_\ell - z)}.$$

Now consider a closed curve γ in the complex plane, whose interior contains the points $\alpha_1, \ldots, \alpha_m$, as well as zero. Applying the residue theorem and

Cauchy's integral formula yields

$$\frac{1}{2\pi i}\oint_{\gamma} F(z)\,dz = \begin{cases} -\sum_{j=1}^{m} \frac{\alpha_j^{s-1}}{\prod_{\substack{1\le \ell\le m\\ \ell\ne j}} (\alpha_\ell - \alpha_j)} & s \ge 1 \\ -\sum_{j=1}^{m} \frac{\alpha_j^{s-1}}{\prod_{\substack{1\le \ell\le m\\ \ell\ne j}} (\alpha_\ell - \alpha_j)} + \frac{1}{\prod_{\ell=1}^{m}\alpha_\ell} & s = 0. \end{cases} \tag{40}$$

On the other hand, since F has no poles outside of γ other than possibly at ∞ (in the case $s \ge m$) we can also express this integral in terms of the residue of F at ∞:

$$\begin{aligned} \frac{1}{2\pi i}\oint_{\gamma} F(z)\,dz &= \operatorname{Res}\left[F(z), \infty\right] \\ &= \operatorname{Res}\left[z^{-2}F(z^{-1}), 0\right]. \end{aligned}$$

We rewrite

$$\begin{aligned} z^{-2}F(z^{-1}) &= \frac{z^{-s-1}}{\prod_{\ell=1}^{m}(\alpha_\ell - z^{-1})} \\ &= \frac{z^{m-s-1}}{\prod_{\ell=1}^{m}(\alpha_\ell z - 1)}. \end{aligned}$$

Cauchy's integral formula then gives

$$\operatorname{Res}\left[F(z), \infty\right] = \begin{cases} 0 & 0 \le s \le m-1 \\ \frac{1}{(m-s)!}\frac{d^{m-s}}{dz^{m-s}}\Big|_{z=0} \frac{1}{\prod_{\ell=1}^{m}(\alpha_\ell z - 1)} & s \ge m. \end{cases}$$

In particular, for $s = m$ we have $\operatorname{Res}[F(z), \infty] = (-1)^m$. Combining with (40) verifies the desired result (39), and moreover, provides a method to calculate similar expressions with $s > m$. □

References

Antal, T. and P. L. Krapivsky (2011). Exact solution of a two-type branching process: models of tumor progression. *Journal of Statistical Mechanics: Theory and Experiment 2011*(08), P08018.

Athreya, K. B. and P. E. Ney (2004). *Branching Processes*. Dover Publications.

Coldman, A. J. and J. H. Goldie (1983). A model for the resistance of tumor cells to cancer chemotherapeutic agents. *Mathematical Biosciences 65*(2), 291–307.

Dewanji, A., E. Luebeck, and S. Moolgavkar (2005). A generalized luria–delbrück model. *Mathematical Biosciences 197*(2), 140–152.

Haeno, H., Y. Iwasa, and F. Michor (2007). The evolution of two mutations during clonal expansion. *Genetics 177*(4), 2209–2221.

Iwasa, Y., M. A. Nowak, and F. Michor (2006). Evolution of resistance during clonal expansion. *Genetics 172*(4), 2557–2566.

Katouli, A. A. and N. L. Komarova (2010). Optimizing combination therapies with existing and future cml drugs. *PloS one 5*(8), e12300.

Komarova, N. (2006). Stochastic modeling of drug resistance in cancer. *Journal of Theoretical Biology 239*(3), 351–366.

Komarova, N. L. (2011, Apr). Mathematical modeling of cyclic treatments of chronic myeloid leukemia. *Math Biosci Eng 8*(2), 289–306.

Komarova, N. L., A. A. Katouli, and D. Wodarz (2009, 02). Combination of two but not three current targeted drugs can improve therapy of chronic myeloid leukemia. *PLoS ONE 4*(2), e4423.

Komarova, N. L. and D. Wodarz (2005). Drug resistance in cancer: Principles of emergence and prevention. *Proceedings of the National Academy of Sciences of the United States of America 102*(27), 9714–9719.

Michor, F., M. A. Nowak, and Y. Iwasa (2006). Evolution of resistance to cancer therapy. *Current Pharmaceutical Design 12*(3), 261–271.
